# Aberrant Development of Speech Processing in Young Children with Autism: New Insights from Neuroimaging Biomarkers

**DOI:** 10.3389/fnins.2016.00393

**Published:** 2016-08-25

**Authors:** Holger F. Sperdin, Marie Schaer

**Affiliations:** ^1^Office Médico-Pédagogique, Department of Psychiatry, University of Geneva School of MedicineGeneva, Switzerland; ^2^Stanford Cognitive & Systems Neuroscience Laboratory, Stanford University School of MedicinePalo Alto, CA, USA

**Keywords:** ASD, MRI, EEG, language development, voice, auditory processing, toddler, infant

## Abstract

From the time of birth, a newborn is continuously exposed and naturally attracted to human voices, and as he grows, he becomes increasingly responsive to these speech stimuli, which are strong drivers for his language development and knowledge acquisition about the world. In contrast, young children with autism spectrum disorder (ASD) are often insensitive to human voices, failing to orient and respond to them. Failure to attend to speech in turn results in altered development of language and social-communication skills. Here, we review the critical role of orienting to speech in ASD, as well as the neural substrates of human voice processing. Recent functional neuroimaging and electroencephalography studies demonstrate that aberrant voice processing could be a promising marker to identify ASD very early on. With the advent of refined brain imaging methods, coupled with the possibility of screening infants and toddlers, predictive brain function biomarkers are actively being examined and are starting to emerge. Their timely identification might not only help to differentiate between phenotypes, but also guide the clinicians in setting up appropriate therapies, and better predicting or quantifying long-term outcome.

## Introduction

Autism, a term initially introduced by Kanner (1943) and almost at the same period by Asperger (1944), is a pervasive disorder of neurodevelopment with an early onset. According to the most recent census, autism affects up to 1 in 68 children (1.5%) in the United States (Baio, 2014). ASD is characterized by impairments in core areas of cognitive and adaptive function, social interactions, and communication (American Psychiatric Association and American Psychiatric Association. Dsm-5 Task Force, 2013). Individuals with ASD show a reduced interest in socially relevant stimuli (McPartland et al., 2011; Pelphrey et al., 2011; Chevallier et al., 2012; Kohls et al., 2012), tend to avoid eye-contact with their immediate surrounding (Senju and Johnson, 2009; Elsabbagh et al., 2012; Jones and Klin, 2013), and show repetitive behaviors and restricted interests (Turner, 1999; Watt et al., 2008; Arnott et al., 2010). Moreover, affected children often present language delay, with deficits in expressive and receptive language skills (Hudry et al., 2010; Eigsti et al., 2011; Mody et al., 2013; Simms and Jin, 2015). Multiple causes are implicated in autism and recent accounts indicate the presence of abnormal development occurring at the cellular and molecular levels during prenatal life (Stoner et al., 2014; Baron-Cohen et al., 2015), with a clear impact of genetic, neurobiological, environmental factors, and combinations thereof (Geschwind and Levitt, 2007; Abrahams and Geschwind, 2010; Betancur, 2011; Zhubi et al., 2014; Robinson et al., 2015). One of the core domains that is particularly impaired and that constitutes a hallmark feature in autism is language. Behaviorally, children with autism do not orient naturally to vocal stimuli as typically developing (TD) children do (Dawson et al., 2004; Kuhl et al., 2005). They often show a reduced sensitivity to human voices, but are responsive to other non-vocal stimuli (Klin, 1991, 1992). This would suggest that the neural mechanisms underlying the orientation to voices and their processing might not develop in the same way as in TD individuals. Currently, the exact time when the developmental trajectory of the brain systems implicated in human voice processing starts to deviate from a normal path is unknown, but recent neuroimaging results that we discuss below suggest the presence of differences already from the age of 2 years (e.g., Lombardo et al., 2015).

Neuroimaging provides an excellent window to better understand the neural bases of speech and language abnormalities in young children with ASD. Differences in brain anatomy have been investigated using structural magnetic resonance imaging (MRI); patterns of changes to structural connectivity have been examined using diffusion tensor imaging (DTI); changes in cortical activity measured using functional MRI (fMRI); and altered spatio-temporal brain dynamics quantified using high-density electroencephalography (EEG). Despite the fact that all these techniques are non-invasive, their use in children involves numerous challenges (de Bie et al., 2010; Raschle et al., 2012). Perhaps the most noteworthy of these challenges is the requirement for the child to remain still for extended periods of time, otherwise creating difficulties for placing the electrode caps on a young child's head in EEG experiments, or leading to movement artifacts in MRI acquisitions. Nonetheless, recent years have seen practical and methodological advancements, which tremendously improved the feasibility of neuroimaging research studies in young children (in particular in the MRI field). For example, mock MRI scanning facilities are increasingly used to prepare preschoolers and school-aged children for a successful real MRI acquisition (Cantlon et al., 2006; Epstein et al., 2007; de Bie et al., 2010; Nordahl et al., 2016). Although initial studies of infants and toddlers used anesthesia or mild sedation (e.g., Boddaert et al., 2004a; Fransson et al., 2007), protocols for obtaining MRI acquisitions during natural sleep have been proposed for young children with ASD (e.g., Nordahl et al., 2008; Ortiz-Mantilla et al., 2010; Eyler et al., 2012; Shen et al., 2013; Lombardo et al., 2015). Scanning during natural sleep allows to study at the functional and structural levels how the brain systems implicated in human speech and language processing are developing in very young infants and toddlers and to detect early biomarkers for ASD. We will discuss how these recent neuroimaging studies performed in very young children with ASD or at risk have begun to reveal impairments at multiple levels in the brain systems implicated in speech and language processing.

**Figure 1:**
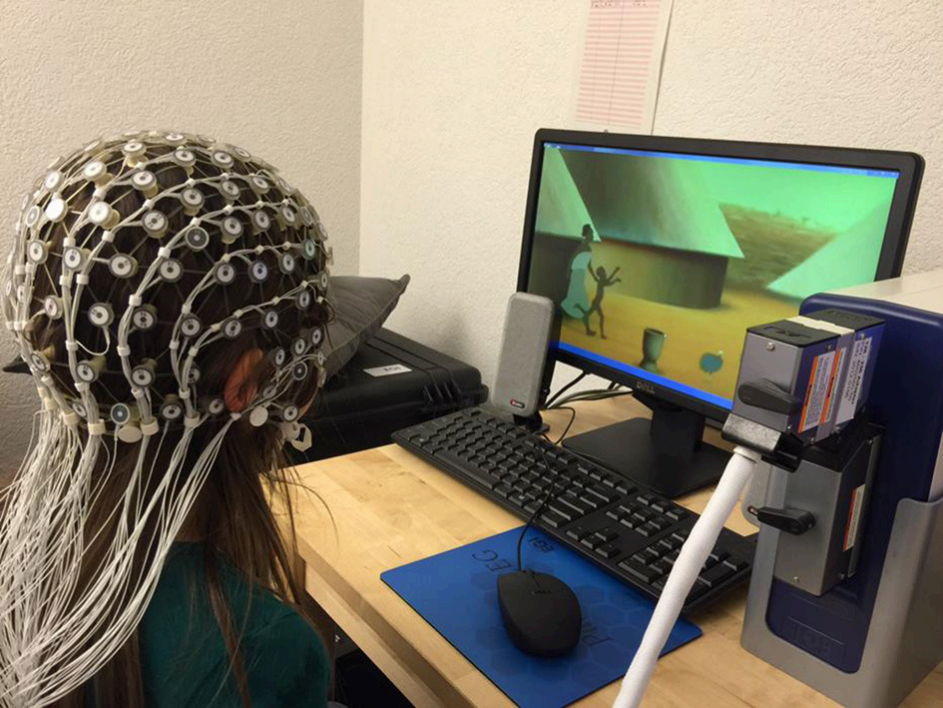
**Example of an EEG set-up with a young child**. A video is displayed on a standard screen, and the child is wearing a 129 electrodes cap. Sounds are displayed via external speakers.

In EEG, the experiments are generally performed during wakefulness (see Figure 1; e.g., Boersma et al., 2013; Kuhl et al., 2013; Seery et al., 2013, 2014). The EEG field has also seen recent methodological advancement. Modern electrical source estimations of high density EEG now reach an approximation in the whole brain of the 3-D distribution of the neuronal activity at each moment in time (Michel et al., 2004; Brunet et al., 2011; Michel and Murray, 2012; Custo et al., 2014) and have been shown to represent stable and reliable estimates when compared with intracranial recordings, lesions and animal studies and other neuroimaging methods (Pittau et al., 2014 for review). For this reason, EEG studies of infants at risk and toddlers with ASD should provide source estimations when possible as they may add valuable information regarding how they differ in their early brain development compared to their TD peers. Improving the precision of source localization by using individual MRI scans of infants/toddlers or age-appropriate template MRIs is also possible. Their estimation in normally and abnormally developing infants/toddlers (or those at risk) can subsequently be compared with results from available fMRI experiments. This is important as most fMRI experiments are currently being performed in a sleep state while EEG experiments are mostly being conducted in awake participants. Finally, compared to the EEG experiments using a traditional voltage waveform analysis approach and that will be reviewed here, electrical neuroimaging methods are reference-independent and take into account the additional information of multichannel electrodes recordings. As such, they avoid the traditional statistical pitfalls inherent to traditional voltage waveform analysis (see Murray et al., 2008 for discussion). So-called “microstate” analyses are also available, allowing to identify dominant state topographies in spontaneous EEG recordings acquired in young infants and toddlers with ASD (Koenig et al., 2002; Lehmann and Michel, 2011). These methods have been successfully applied on EEG data acquired in clinical population of children and young adolescents (Rihs et al., 2013; Berchio et al., 2014; Tomescu et al., 2014) and could be used to examine the developmental trajectories of infants/toddlers with ASD and infants at risk for autism.

While other reviews of auditory and speech processing impairments have been published focusing on older children and adults (e.g., Haesen et al., 2011; Kujala et al., 2013), here, we provide an overview of some of the most recent neuroimaging experiments (primarily fMRI and EEG) of very young children (before 4 years of age) with ASD investigating impairments in the brain systems implicated in human vocalizations and henceforth speech and language processing (e.g., Kuhl et al., 2013; Lombardo et al., 2015) and in at-risk populations (before the age of 2) to identify early endophenotypes (Seery et al., 2013, 2014; Blasi et al., 2015). We will principally review recent experiments that have used voice related auditory stimuli (e.g., sentences, words, syllables). After a brief description of the language development in the typically developing individual during the first year, we will summarize part of the clinical body of evidence pointing to altered speech and language development in very young children with ASD and in infants at high-risk for ASD. Afterwards, we describe the neural systems within the superior temporal cortical regions implicated in human voice processing and their development in the TD brain and present functional evidence in adults and young adolescents indicating the presence of an aberrant form of voice processing. Next we focus on the cross-sectional studies using fMRI and EEG which were conducted at specific time points during infancy/toddlerhood. They address group differences in early speech and language-related processing within these voice areas within superior temporal cortical regions along other language related brain neural systems. The results thereof indicate the presence of structural, functional, and connectivity group differences being already present in infancy and/or toddlerhood. We finally highlight recent results from the few existing prospective fMRI and EEG studies which employed a longitudinal design and demonstrated by using voice related auditory stimuli that aberrant voice processing is not only a feature present in older children and adults with ASD but also a promising candidate to identify ASD very early on during the development.

## Language development in typically developing individuals

**Figure 2:**
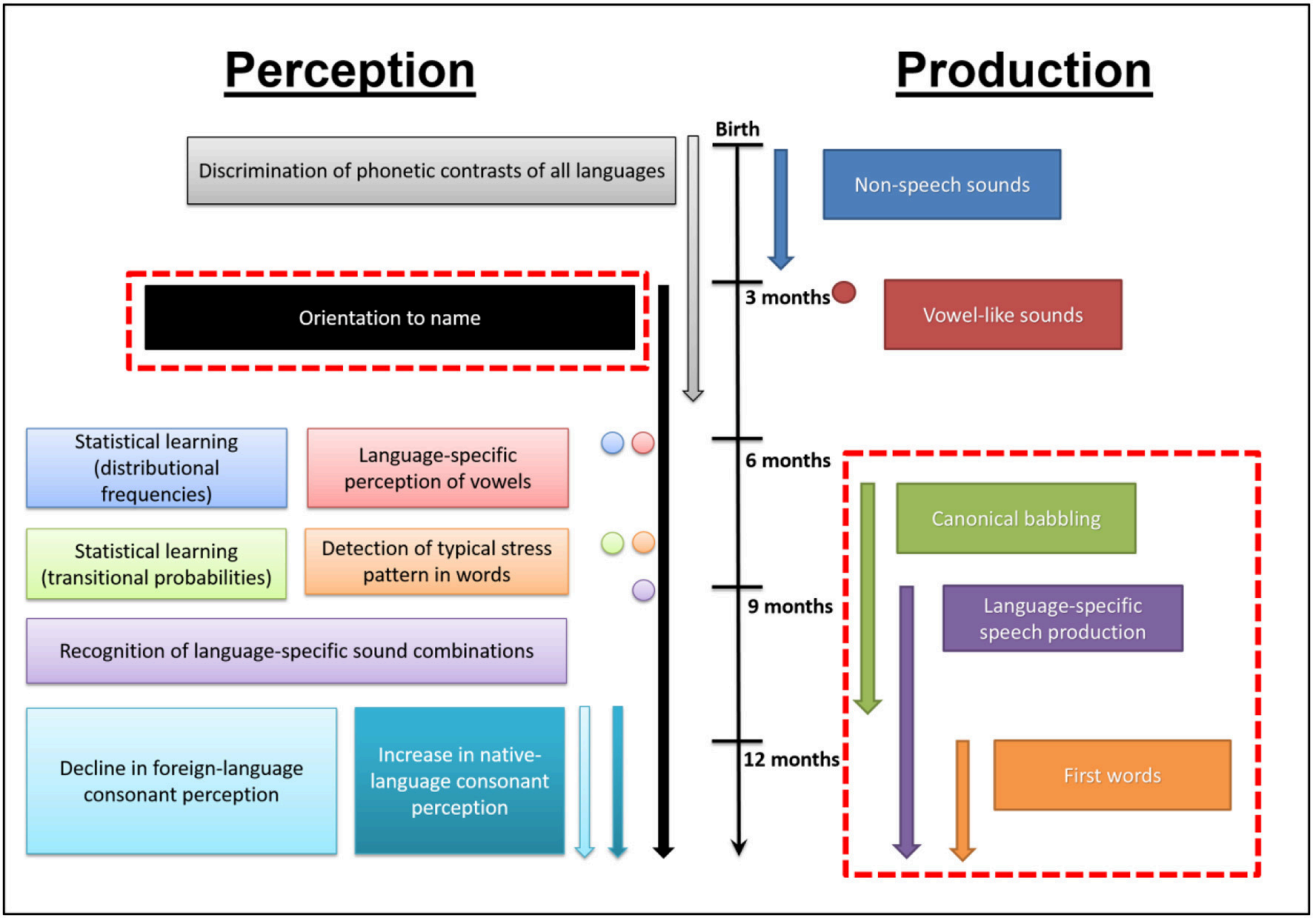
**Illustration of the changes occurring in speech perception and production in typically developing human infants during their first year of life (adapted from Kuhl, 2004)**. Red dashed rectangles indicate early expressive and receptive language delays/impairments (that is, unresponsiveness to name, delayed canonical babbling, increased non-speech productions, decreased speech-like vocalization, delayed occurrence of the first words) known to be sensitive indicators of an increased risk for later being diagnosed with ASD

The different steps involved in early speech perception and production have been extensively examined (see Figure 2; Kuhl, 2004, 2010 for reviews). TD newborns are rapidly attracted by human voices within the first days of life (Cheng et al., 2012). At 1 month of age, they are already responsive to speech sounds (Eimas et al., 1971). Language-related brain areas are activated in response to human speech sounds to some extent in 3 month old infants, well before the onset of speech production (Dehaene-Lambertz et al., 2002, 2006), while cerebral specialization for the human voice over other sounds emerges over the first 6 months of life (Minagawa-Kawai et al., 2011; Lloyd-Fox et al., 2012). By 4 months, infants know that speech conveys information that relate words to physical objects (Marno et al., 2015). Around 5 months, they can recognize the sound patterns of their own name, and between 6 and 9 months they are capable of correctly directing their gaze to named pictures suggesting the presence of some form of word comprehension (Mandel et al., 1995; Tincoff and Jusczyk, 1999, 2012; Bergelson and Swingley, 2012). With respect to pre-linguistic production skills, between 0 and 2 months, newborns first produce vegetative vocalizations (non-speech sounds such as burps, coughs, and cries). At 3 months, infants start to produce vowel-like sounds followed by the onset around 6 months of a babbling phase that becomes robust by 10 months of age. Canonical babbling is a precursor to the emergence of the first words production, which are generally produced by the end of the first year. During the first year of the infant's development and the following years into toddlerhood, the human voice is a natural driver for the infant's language skills development.

## Language development in toddlers with ASD and infants at risk

In individuals with autism, the degree of impairment and delay in language greatly varies from one person to another, with a tremendous heterogeneity in early language development and later clinical outcomes (Mitchell et al., 2006; Geurts and Embrechts, 2008; Luyster et al., 2008; Tager-Flusberg et al., 2009; Lenroot and Yeung, 2013; Lord et al., 2015). Some toddlers present substantial delay or deficits while others have a typical early language development or mild delay and catch up. The former are children that often show the most severe and pertaining symptoms in the long term, compared to those with relatively preserved language abilities (Fein et al., 2013; Kasari et al., 2013; Tager-Flusberg and Kasari, 2013). Converging clinical estimates indicate that more than half of the children with ASD will have persisting language impairments throughout their lifespan (e.g., Anderson et al., 2007; Pickles et al., 2014). So far, the heterogeneity of early language development and the neurodevelopmental basis for this variability in clinical outcomes are not fully understood. In this review, we also discuss how neuroimaging studies examining the neural bases of early human speech and language impairments in autism have started to be used to predict outcome in affected children.

In toddlers with ASD, language difficulties are often present both when they are spoken to (i.e., receptive language) and when they express themselves (i.e., expressive language; Hudry et al., 2010; Simms and Jin, 2015). Indeed, parents of children diagnosed with ASD often sought medical advice because of strong concerns related to language development (Dahlgren and Gillberg, 1989; De Giacomo and Fombonne, 1998; Wetherby et al., 2004; Herlihy et al., 2015). Retrospective interviews with the families, analysis of retrospective video birthday tapes recorded at 12 months of age of children diagnosed with ASD, as well as prospective accounts of infants at risk often report an unresponsiveness to name and a general lack of orientation to human voices (Osterling and Dawson, 1994; Baranek, 1999; Yirmiya et al., 2006; Nadig et al., 2007; Oner et al., 2014; Stenberg et al., 2014). In sum, a large body of clinical studies to date point to expressive and receptive language deficits already in the first year of life for young children who will subsequently develop ASD during toddlerhood, suggesting that the neural systems responsible for orienting to and processing human vocalizations are altered very early on.

Infants at risk for autism are increasingly studied prospectively, to measure whether the abilities to understand language described above are already altered in infants who will develop autism later on. Siblings of a child with ASD have a very high risk to develop ASD, ~20 times higher compared to infants with no family history of ASD (Rogers, 2009; Ozonoff et al., 2011). In a context where early intensive non-pharmacological interventions are critical to improve the long term outcome of affected individuals (Dawson, 2008; Dawson et al., 2010; Klintwall et al., 2013), it is instrumental to detect ASD as early as possible, (see also Schaer et al., 2014 for a review). As such, studies of high-risk infants allow to map the early developmental trajectories of infants who will develop ASD, and to highlight endophenotypes of ASD (Viding and Blakemore, 2007). Numerous studies of high-risk infants focused on early language development as delays in communication and language development become apparent early in life, even before the first year or shortly thereafter. Differences in vocal production (such as consonant inventory, presence of canonical syllables, and non-speech vocalizations), between low-risk and high-risk infants between 9 and 12 months has been associated with later outcomes at 24 months (Paul et al., 2011; Jones et al., 2014 for review). Another prospective study of infants at risk tested at target ages 6, 14, and 24 months and who were followed up and diagnosed with ASD at 24 months indicates that language delays or deficits are already observable at around 14 months of age (Landa and Garrett-Mayer, 2006). A recent retrospective study of toddlers with ASD reported low rates of canonical babbling and vocalization frequency between 9 and 12 months and 15–18 month compared to age-matched TD peers, several months before a diagnosis of ASD was made (Patten et al., 2014). As a result, a delay or deficit in language development very early on has become integral part of the red flags indicating a greater susceptibility for developing autism (Barbaro and Dissanayake, 2009; Zwaigenbaum et al., 2013; see Figure 2, red dashed rectangles).

## The voice as a unique auditory stimulus in the typically developing brain

**Figure 3:**
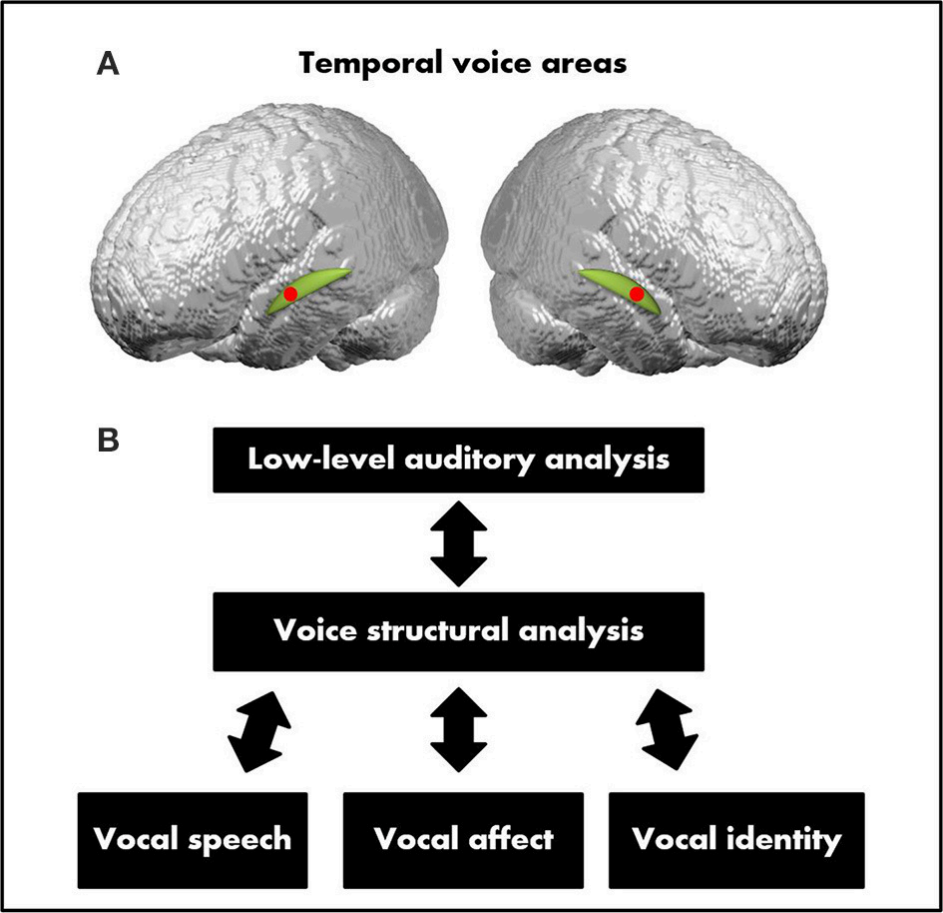
**(A)** Temporal voice areas (TVA). The TVA (represented here by the red dots) are mostly located along the middle and anterior parts of the superior temporal sulcus (STS) bilaterally over the temporal plan (represented here in green). **(B)** A model of voice perception. After a stage of voice structural encoding constrained to vocal sounds, three partially dissociable functional pathways process the three main types of vocal information: speech, identity, and affect (adapted from Belin et al., 2004).

The human voice is clearly one of the most salient and important auditory stimuli in our acoustic environment. It conveys both linguistic and extra-linguistic information. It delivers speech information permitting us as individuals to recognize the others and to attribute emotional states to them (Belin et al., 2000; Ethofer et al., 2009). While language is generally thought to be processed in specialized brain areas such as the inferior frontal gyrus (IFG; also known as Broca's area), the superior and middle temporal gyruses (STG, MTG, also termed Wernicke's area) and angular gyrus, voice selective areas have been located bilaterally in the upper bank of the middle superior temporal sulcus (STS) over the temporal poles (see Figure 3A). Their existence has particularly been highlighted by fMRI experiments in the adult brain by comparing the cortical activation patterns induced by vocal vs. non-vocal sounds (Belin et al., 2000, 2004; Kriegstein and Giraud, 2004; Belin, 2006, for review; Latinus and Belin, 2011; Deen et al., 2015; Pernet et al., 2015). When a voice is perceived, the brain begins by a low-level analysis of the acoustic features involving sub-cortical nuclei and primary auditory cortical regions. Subsequently, the voice is processed during a voice-specific stage where its structure is encoded. Three types of vocal information are then extracted and further processed in partially separable but functionally interacting pathways: the speech content, the affective content and the vocal identity (see Figure 3B). Early studies of very young infants have shown that voice sensitive cortices within the temporal areas develop as a voice selective brain system between 4 and 7 months of age in the typically developing brain and to become responsive to the quality of the voice during speech (emotional voice prosody) by the age of 7 months with a right hemispheric dominance (Belin and Grosbras, 2010; Grossmann et al., 2010; Blasi et al., 2011; Lloyd-Fox et al., 2012). A recent study of typically developing preschoolers (mean age = 5.8 years) compared voice-specific vs. speech-sound specific functional brain activity and demonstrated that the right STS already works as a specialized temporal voice system (Raschle et al., 2014), similarly to what has been reported in the adult brain (Belin et al., 2002; Belin and Zatorre, 2003; von Kriegstein et al., 2003).

## Aberrant voice processing in older children and adults with ASD

Difficulties to speak and to interact socially in an appropriate manner are central traits of autism and have been linked to abnormal processing of social information in both the visual and auditory modalities (e.g., Dawson et al., 1998, 2004; Klin et al., 2009; Chevallier et al., 2012 for review). Children and adults with ASD often tend to ignore human vocalizations in their surrounding but are responsive to other non-vocal stimuli indicating a detachment from their social environment (Klin, 1991, 1992; Kuhl et al., 2005). Early acquisition of language capacities is closely intertwined to social function in typically developing children and children on the spectrum (e.g., Goldstein et al., 2003; Kuhl et al., 2003; Norbury et al., 2010). Although, it is still not established why many individuals with ASD are often insensitive to human vocalizations, anomalies within voice selective areas have been highlighted in older children and adults with ASD. Using fMRI, Gervais and colleagues showed in a seminal study that adults on the spectrum (mean age = 25 years) failed to activate voice selective regions of the STS but showed similar activation patterns to the comparison group in response to non-vocal sounds (Gervais et al., 2004). This finding suggested an aberrant form of processing with respect to auditory information having a social content such as a voice does. This would suggest that a sound with a social content might not be adequately processed, most likely due to the abnormal development of cerebral regions implicated in the analysis of the social content of auditory stimuli (Gervais et al., 2004). Decreased gray matter volume in the bilateral voice specific STS have been observed in 10 year old children with ASD (Boddaert et al., 2004b). Abrams and colleagues hypothesized that this may be the consequence of individuals with ASD having impaired function of emotional and reward systems which in turn prevents them from engaging with acoustic information with a high social content such as speech stimuli (Abrams et al., 2013). In order to test their hypothesis, Abrams and colleagues scanned and compared 20 young children with ASD to age- and intelligence quotient-matched TD controls (mean age = 9.8 years) using a resting-state fMRI protocol. By looking at the intrinsic functional connectivity in the voice-selective posterior STS (pSTS) bilaterally, they found the presence of underconnectivity between the left-hemisphere pSTS and the bilateral ventral tegmental areas in children with ASD. This was also the case for other regions such as the nucleus accumbens, left-hemisphere insula, orbitofrontal, and ventromedial prefrontal cortices. Moreover, diminished connectivity was evident between right-hemisphere pSTS and the orbitofrontal cortex and amygdala.

An important aspect of the study by Abrams and colleagues was that the degree of underconnectivity between voice-selective cortex and reward pathways predicted symptom severity for communication deficits in children with ASD, thus providing a connectivity biomarker for this specific group of patients. The study of the connectivity profile using a resting-state fMRI protocol during infancy and toddlerhood is now needed to further our understanding regarding how the functional connectivity between reward pathways and voice- and speech-related brain areas develops. Several neuroimaging studies performed in high-functioning older children and adults with ASD point to the presence of impairments in the neural basis of language processing in general (e.g., Gaffrey et al., 2007; Knaus et al., 2008; Herringshaw et al., 2016 for recent review). These experiments often including an overt task have revealed the existence of abnormal frontal and/or temporal responses during language processing tasks compared to TD individuals and reversed or reduced laterality within fronto-temporal language regions (e.g., Boddaert et al., 2003; Flagg et al., 2005; Kleinhans et al., 2008; Knaus et al., 2010; Lindell and Hudry, 2013; Herringshaw et al., 2016). In sum, experiments that used language tasks or rest scanning in the awake state indicate the presence of aberrant processing of human vocalizations in older children and adults with ASD.

In the following sections, we summarize the experiments investigating the neuronal underpinnings of human speech and language abnormalities during early periods of development: infancy and toddlerhood. These recent experiments have mostly used auditory speech stimuli which implicitly require analysis of the human voice. Importantly, several studies have included low-functioning toddlers with ASD or studied infants at high-risk for ASD. In contrast to experiments performed with older high-functioning children and adults with ASD and that include a task, scanning of very young infants is performed during natural sleep, at rest in the absence of an overt task or by passively presenting speech-stimuli (e.g., sentences, words, syllables). Overall, findings from these studies indicate that the brain systems implicated in human speech and language processing follow a different developmental pathway in individuals with ASD when compared to TD individuals very early on in the development already. We begin by a summary of the structural and functional differences that have been found in young toddlers with ASD and infants at risk.

## Structural differences in toddlers with ASD and infants at risk

Several studies suggested brain overgrowth during the first year of life in toddlers with ASD (e.g., Courchesne et al., 2001, 2003, 2011; Redcay and Courchesne, 2005; Hazlett et al., 2011; Nordahl et al., 2011; Shen et al., 2013). For example, Sparks and colleagues measured an increased brain volume in toddlers with ASD (aged 3–4 years) compared to aged-matched TD controls and developmentally delayed (DD) children (Sparks et al., 2002). Longitudinal measurements indicate the presence of gray and white matter cerebral overgrowth in toddlers at 2 years (Hazlett et al., 2005). In a longitudinal study, Schumann and colleagues followed up toddlers and school aged children with ASD (1.5 years up to 5 years of age). They found both gray and white matter enlargements by 2.5 years of age in fronto-temporal regions along with cingulate cortices, regions related but not limited to language development (Schumann et al., 2010). A global increase in gray matter volumes in toddlers with ASD aged between 2 and 3 years compared to DD toddlers has recently been reported (Xiao et al., 2014). The locus of this difference manifested regionally in the right STG, a cortical region known to be involved in spoken language comprehension (Lattner et al., 2005). In older children with ASD, this locus has been shown to be enlarged to aged match controls (mean age = 13.5 years; Jou et al., 2010) and to exhibit a different pattern of activation during speech processing compared to TD adolescents (mean age = 12 years; Lai et al., 2011). Other studies indicate the presence of structural anomalies within language-related brain areas in older children and adults (e.g., Prigge et al., 2013; Itahashi et al., 2015; Lai et al., 2015). It is hypothesized that this early brain overgrowth during infancy and toddlerhood in ASD is followed by a period of decline in brain size from childhood to adulthood (Courchesne et al., 2011; Lange et al., 2015).

Diffusion imaging studies have also revealed the presence of widespread disruption of white matter integrity in long-range and short-range connections in toddlers, older children and adults with ASD (see Hoppenbrouwers et al., 2014; Conti et al., 2015 for reviews). For example, accelerated white matter maturation has been reported in a small sample of seven participants aged between 1.8 and 3.3 years (Ben Bashat et al., 2007). Altered white matter integrity has also been found in toddlers with ASD (mean age = 3.2 years; Weinstein et al., 2011). Xiao et al. (2014) showed altered structural brain connectivity in multiple regions that have been related but not limited to language functions such as the posterior cingulate cortex, subregions of the limbic lobes as well as the corpus callosum in toddlers with ASD aged between 2 and 3 years (Xiao et al., 2014). It even appears that white matter anomalies might develop before the first year in infants who are later diagnosed with ASD. Wolff and collegues observed abnormalities in white matter fiber tracts in infants at risk (at 6 months) and who were diagnosed at 24 months (Wolff et al., 2012). In a recent study of infants at high-risk for ASD and diagnosed at 24 months, white matter connectivity abnormalities were present specifically over Broca's area in the frontal lobes, and more generally in the temporal, parietal, occipital lobes as compared to both low-and high-risk infants not classified as ASD (Lewis et al., 2014).

Anatomical data indicate a period of early brain overgrowth (between 1 and 5 years) followed by normalization during adolescence. Structural connectivity experiments also report a developmental shift from greater structural connectivity in very young children with ASD to lower connectivity in older children (see Hoppenbrouwers et al., 2014; Conti et al., 2015 for recent discussions). These alterations impinge on acquiring normal language functions and lead to other higher-order cognitive, social, and communicative functions deficits. However, it is still unclear how those differences relate and can yet reflect the early language development heterogeneity inherent to autism. The field is also currently hampered by the widespread methodology differences in terms of subject inclusion criteria (high vs. low functioning autism), control groups, size of cohorts, age range, neuroimaging methods and parameters. Future cross-sectional and longitudinal experiments spanning early childhood to adulthood are necessary including larger sample size. Only then it will be possible to get a clearer picture within this complex and increasingly expanding field of research.

## Functional studies in toddlers with ASD using voice-related auditory stimuli

Brain abnormalities underlying human voice processing during toddlerhood have also been found. A seminal EEG experiment performed by Kuhl et al. (2005) indicated the presence of different event-related potential (ERP) response pattern to speech stimuli in toddlers with ASD compared to TD (mean age = 3.5 years). In this experiment, toddlers were passively presented with standard and deviant phonemes. In individuals with typical development, contrasting the brain responses that are produced by the deviant sound with the ones produced by the standard sound causes a mismatch negativity (MMN). The MMN is a robust index of automatic sound discrimination (MMN, Näätänen, 1995, 2003 for reviews). In toddlers with ASD, there was no evidence for an MMN, whereas in TD the MMN was present. Using an auditory preference test, the group of toddlers with ASD was subdivided between those who preferred human vocalization (i.e., motherese speech sounds) and those who preferred non-speech analogs. An MMN appeared in the group that preferred motherese sounds similarly to what was found in TD toddlers while toddlers with ASD who preferred the non-speech sounds still did not exhibit an MMN. These findings are important as they reveal a link between early social preferences and early language processing skills in toddlers with ASD.

A functional study by Redcay and Courchesne (2008) found that the brain systems of speech perception were responding differently to speech stimuli in toddlers with ASD compared to TD toddlers. In a cross-sectional experiment with a small sample size, the authors scanned toddlers aged between 2 and 3 years using a natural sleep fMRI experimental design. They recorded brain activity when toddlers were asleep and listened to normal speech of a human voice (forward and backward speech stimuli), and the ones from toddlers with ASD. Results indicated the recruitment of different regions and with a different laterality dominance. Specifically, toddlers with ASD exhibited hypoactivation of many regions traditionally recruited during early language acquisition in comparison to those with TD, suggesting that at that age toddlers with ASD are already on a deviant developmental trajectory characterized by a greater recruitment of right hemispheric regions during speech perception (Redcay and Courchesne, 2008). The same research group then performed an fMRI experiment where they increased their sample size and included even younger children (aged between 12 and 48 months; Eyler et al., 2012). Using a prospective, cross-sectional design this time, 80 toddlers listened to a bedtime story during their sleep (speech sound stimuli from a human voice). Toddlers were also followed up to get record of their evolution and to ensure confirmation of later ASD diagnosis. A different brain pattern of brain activation was found between toddlers who were at risk and who were later diagnosed with ASD and the toddlers who followed a normal development path. Specifically, toddlers with ASD had deficient left hemisphere responses to speech sounds with an abnormal right-lateralized temporal cortical response to language; this deficit worsened during growth to become most severe by the age of four. Contrarily, TD toddlers had a reversed pattern of brain activity and lateralization with the presence of more temporal cortical responses and a left lateralized pattern of brain activation that became stronger with development. Two important observations can be made. Firstly, lateralization in response to auditory speech sounds differs between groups. Abnormal lateralization in response to language has also been reported in experiments performed in older children and adults with ASD (e.g., Kleinhans et al., 2008; Minagawa-Kawai et al., 2009). Secondly, weaker brain responses to speech sounds over the temporal pole were present in the ASD group, suggesting the presence of an early specific and abnormal brain response pattern to speech sounds in toddlers with ASD (Eyler et al., 2012).

## Functional studies in infants at risk using voice-related auditory stimuli

In a recent study, Seery et al. (2013) suggested that this atypical lateralization is an ASD endophenotype already observable during the first year of life. Comparing infants at risk for ASD with low-risk infants aged between 6 and 12 months, the authors reported significant group differences in the development of lateralized ERP responses to speech (using consonant-vowel auditory stimuli). More specifically, the low-risk group displayed a lateralized response to the speech sounds whereas the high-risk group did not (Seery et al., 2013). In a subsequent study, the same research group found atypical ERPs to repeated speech sounds in 9 month old infants at risk (Seery et al., 2014). Atypical lateralization of the ERP to words at 2 years of age has also been observed in ASD toddlers with poor social skills (Kuhl et al., 2013). In a very recent sleep fMRI study that also included behavioral assessment of parent-infant interactions, Blasi et al. (2015) compared cortical responses between emotional voices and environmental sound stimuli. They found that high-risk infants for ASD (aged between 4 and 7 months) did not show this early specialization suggesting the presence very early on of atypical neural responses to human voice with and without emotional valence in at-risk populations, at least (Blasi et al., 2015). Taken together, these results indicate that during early development, speech, and language-critical areas over the temporal poles do not show the same brain responses to voice related stimuli as observed in TD young individuals. An absence or atypical lateralization of brain responses and functional hypoactivation in response to speech related content were already reported in infants at risk and toddlers with ASD. Moreover, voice-selective cortices in populations show lesser degree of specialization already in infants at risk. In sum, similarly to the experiments performed in high-functioning older children and despite the use of different experimental conditions, abnormalities in lateralization and aberrant functional activation during voice processing are already found very early on, during infancy and toddlerhood.

## Dysfunctional connectivity in toddlers with ASD and infants at risk using voice-related auditory stimuli

Aside from the differences in functional activation described in the previous section, extant functional connectivity studies using fMRI or EEG with voice-related stimuli have shown altered connectivity between brain regions involved in language processing in young children with autism. Using fMRI, Dinstein and colleagues demonstrated reduced inter-hemispheric synchrony across language brain areas in young toddlers with ASD (Dinstein et al., 2011). They recorded spontaneous brain activity in three groups of toddlers during natural sleep (TD, ASD, and language delay). Seventy-two participants aged between 12 and 46 months (mean age = 29 months for the toddlers with ASD) were presented with auditory stimulation containing words, pseudo words, sentences, tones, or environmental sounds. The aim was to test for differences in synchronization between various brain regions between the three groups and explore a possible relation with the development of early autistic behavioral symptoms within the group of toddlers with ASD. Stimulus-evoked responses were regressed out so as to only keep spontaneous fMRI fluctuations in the data. In doing so, the authors controlled that any differences in synchronization between the groups were not due to differences in auditory-evoked responses between participants. Results indicated the presence of weaker interhemispheric synchronization between the IFG and the STG, two brain areas mediating speech and language processing in the ASD toddler group as compared to the two other groups. Moreover, analyses within the group of toddlers with ASD revealed that the synchronization strength was highest in those with overall good verbal capacities and was weakest in those with impaired verbal capacities. This indicates that, the functional connectivity between regions implicated in language processing has a different pattern compared to TD peers in toddlerhood already (Dinstein et al., 2011).

Using EEG, aberrant reduced functional connectivity has even been reported before the onset of any ASD symptoms, in infants at risk for ASD (Righi et al., 2014). They presented speech sounds (syllables) in the awake state, while EEG was concomitantly acquired in infants at high-risk and infants at low-risk for ASD. Acquisitions were performed at 6 and at 12 months of age. The participants were followed up at 36 months in order to identify which ones would develop ASD or not. By computing the intra-hemispheric linear coherence in the gamma frequency band, (that is, an estimation of synchronization across brain regions) between electrodes of interest located over the frontal and temporo-parietal regions in the left and right hemispheres, the authors found that at 6 months, linear coherence values were similar across groups. At 12 months however, infants at high-risk and later diagnosed with ASD showed reduced functional connectivity (that is, lower linear coherence, and thus less integration) compared to both infants at low-risk and those at high-risk who were not later diagnosed with ASD. In addition, significant differences in functional connectivity between the low-risk and high-risk infants who did not become autistic were found, with lower coherence values in the high-risk infant group. In contrast to what has been previously reported in fMRI with toddlers (Redcay and Courchesne, 2008; Eyler et al., 2012) or with EEG in infants at-risk for ASD (Seery et al., 2013), the study did not reveal any early group differences in hemispheric lateralization. However, as discussed by the authors, the estimation of the linear coherence is an approach less sensitive to stimulus-locked activity whereas fMRI and ERPs are. As such Righi's approach might not have captured existing hemispheric differences. Taken together, published functional connectivity studies examining the early development of language related brain areas demonstrate aberrant brain connectivity patterns in both toddlers with ASD (Dinstein et al., 2011), and in 12 month old infants at risk who later develop autism (Righi et al., 2014). This suggests that aberrant wiring of the cerebral regions responsible for language processing precede the onset of the typical autism phenotype, and might be responsible for the early signs clinically observed in infants who will develop ASD, such as unresponsiveness to name, lack of orientation to human voices and delay in the development of receptive and expressive language skills.

## Language heterogeneity in toddlers with ASD: insights from early brain biomarkers

Findings reviewed so far indicate early functional differences in speech-related processing within superior temporal cortical regions and other language-critical brain areas. The anatomical and connectivity differences reported above in toddlers with ASD and infants at risk for ASD mostly correspond to group differences. While these results are informative, they do not permit to fully address the critical question of the heterogeneity of early language development in ASD and its relation to later outcome. To successfully tackle the question of heterogeneity, research groups have started using prospective and longitudinal designs with larger sample sizes to examine the hypothesis that different subgroups of individuals with ASD have different phenotypes and developmental pathways (Kuhl et al., 2013; Lombardo et al., 2015). These studies open avenues to better understand predictor of outcomes, as predictive brain biomarkers that can differentiate between these subtle phenotypes emerge (Bölte et al., 2013; Lenroot and Yeung, 2013; Ecker et al., 2015). For instance, in a recent developmental study, Lombardo et al. (2015) searched for early functional neuroimaging biomarkers that would reflect the heterogeneity observed in early language development in ASD. The authors measured early cortical responses to speech using a prospective sleep fMRI paradigm (participants aged between 12 and 48 months). The experiment included four different groups with TD infants, infants with a language/developmental delay (LD/DD), infants with ASD having a good language outcome at 4 years of age (“ASD good”) or a bad language development outcome (“ASD Poor”; that is, they measured developmental trajectories of language growth over the first 4 years of life). Pre-diagnosis fMRI brain data in response to three types of speech stimuli (complex forward speech, simple forward speech, and backward speech) were acquired in each participants. The aim was to test whether early functional measures could have a predictive value when combined with clinical-behavioral information. First, they found that in response to speech stimuli, “ASD good” toddlers recruited language-sensitive superior temporal cortices in a very similar way to the control groups (that is, the non-ASD language/developmentally delayed individuals). However, in the “ASD poor” group, language-sensitive superior temporal cortices were found to be hypoactive in response to the same speech stimuli. The multivoxel activation pattern was different to the one observed in the three other control groups indicating a lack of functional differentiation to these speech stimuli in the ASD poor group. Another important finding by Lombardo et al. (2015) was that the brain response patterns to general auditory processing was preserved in the “ASD poor” group (that is, similar to the other control groups), whereas the brain activity specifically related to language and speech was weaker and less specific. For example no engagement of the left hemisphere was found as this was the case in the three other groups. This would suggest on the one hand a general preservation of the neural systems devoted to general auditory processing during infancy and on the other, the presence of a dysfunction of the neural systems at a higher level of processing and implicated in voice-related content leading to the aberrant processing of auditory stimuli containing speech and language related information. Interestingly, the connectivity between primary auditory cortex and the reward and affective brain circuitry seems to be preserved in high-functioning older children with ASD, whereas connectivity between the voice structural module (see Figure 3B) described in Belin's model above and the reward system is impaired, preventing the normal processing of speech related content but allowing low-level sensory processing to occur (Abrams et al., 2013).

The results provided by the study by Lombardo et al. are important for several reasons. First, they indicate the presence during toddlerhood already of brain related differences in the neural underpinnings implicated in the processing of early voice related auditory information and this between different subtypes of ASD (that is, differences between ASD poor and ASD good). Second, longitudinal measurements of pre-diagnostic clinical behavioral information and early fMRI language and speech-related brain responses and combination thereof was found to have a strong predictive value in terms of determining later ASD subgroup prognosis.

Other experiments also point to the presence of early brain biomarkers in EEG. Kuhl et al. (2013) found that the response pattern of ERPs to words at the age of 2 year was predictive of later receptive language capacities at ages 4 and 6 years (Kuhl et al., 2013). In comparison to the Lombardo et al. (2015) study where clinical groups were subdivided based on their language skills, Kuhl and colleagues compared the ERPs in response to words in 2 year old children that were subdivided as a function of social symptoms, into “ASD high” (sever social symptoms) and “ASD low” less social symptoms. Results showed only a left lateralized brain response similar to the TD group in the “ASD low” group. Only the single electrode where the time locked response manifested was different between those two groups (T3 for TD and P3 for “ASD low”). For the “ASD high” group on the other hand, the ERP was more diffuse and right lateralized. Then, in a second phase of the study, the authors looked at the predictive power of their P3 effect found in the first phase for all ASD toddlers on linguistic, cognitive, and adaptive functions at ages 4 and 6 years. For the ASD toddlers who had strong negativity in the ERP to known words at P3 measured at enrollment, a better outcome was observed at 6 years of age. In stark contrast, toddlers with ASD who did not show this ERP sensitivity at intake had worse outcomes (that is, they showed less improvement). Interestingly, the ERP measures to words furthermore exceeded the predictive value of cognitive measures performed at intake. Another important finding by this study was that the predictive aspect of the brain response to words at age 2 years of age did not modulate depending on the type of intensive treatments the toddlers received. Adding another control condition with no treatments may perhaps have added valuable information with respect to the effectiveness of the treatments as recent evidences indicate that early behavioral intervention is associated with normalized patterns of EEG brain activity in the visual modality at least (Dawson et al., 2012). Unfortunately and as mentioned by the authors, the source localization of those differences could not be performed. As already discussed, methods for estimating inverse solutions are now available and it will be important in further studies to include those when possible.

## Perspective and future directions

Neuroimaging studies using exciting new approaches that combine early brain measurements with early behavioral data are starting to highlight important differences in the functional and structural wiring of the young autistic brain compared to the TD one using auditory speech and language related stimuli. With time, the field will hopefully see the appearance of other experiments with larger sample sizes combined with longitudinal measurements to subtype ASD infants according to the core impaired dimension of early language development that was the main focus of the present review, and of social interaction and communication deficits that are both central hallmarks of autism. This will help to explore the efficiency of early intervention in correcting the developmental trajectories on the one hand, and to tackle the question of heterogeneity inherent to autism on the other. Ultimately this will lead to the possibility of improving, developing and modifying therapeutic interventions and adapting them depending to the infant's specific needs. Moreover, because autism cannot always be diagnosed with high certainty before the age of 2–3 years (Zwaigenbaum et al., 2009; Jones et al., 2014), additional prospective longitudinal studies of high-risk populations are necessary to provide understanding about how, when and where developmental trajectories that result in ASD deviate from the TD young brain. These later will in turn aid to understand the general heterogeneity observed in early language development in autism as well as allow early identification of toddlers who should receive intensive therapeutic intervention. The few experiments of infants at risk reported here indicate that brain differences in response to human voice and its speech and language related content are already present before the end of the first year in some cases suggesting that aberrant voice processing could be a promising marker to identify ASD very early on.

Achieving a better understanding regarding which neural systems implicated in speech and language-critical processing are impaired very early on is difficult to highlight and this for several reasons. First, scanning individuals aged between 6 months and 3 year implies using scanning conditions with passive presentation of stimuli most of the time. Implementing tasks that need an engagement of the participant at such a young age is difficult to achieve. For example asking what is voice specific vs. speech specific has only been addressed by one study so far, which used a behavioral task in preschool TD children aged around 6 years (Raschle et al., 2014). In the studies discussed in this review, based in infants and toddlers (i.e., before 4 years), the vast majority of experiments used contrast between speech stimuli and rest, reversed speech or other auditory stimuli (words, sentences, and syllables). Yet, contrasting speech stimuli vs. rest activation does not allow to determine what is specifically related to the voice and what is related to the speech content. The question remains open as to whether a voice specific impairment is present in the brain of young children with ASD. Thus, future experiments should include contrasts between voice specific vs. speech specific brain related activity. This would allow to test whether aberrant voice processing that has been reported in older children and young adults is indeed a hallmark in autism very early on leading to impairments in the social-communication language brain. Currently there is only one experiment to our knowledge where the brain responses to human vocalizations alone were contrasted with non-vocal sounds in infants at risk for autism (Blasi et al., 2015). However, the authors reported that their sample had not been assessed for ASD at the age of 3 years yet. Further work is thus needed to resolve this issue.

Some authors have also recently hypothesized that it is not human speech *per se* that is an issue in autism but rather the mode of communication of speech that might be challenging for individuals with ASD. For example, recent results suggest that the mode of presentation of human speech sounds might play a role in speech perception in older children and young adults with autism. In an fMRI experiments, Lai et al. (2012) passively presented familiar human speech stimuli (spoken sentences by parents) or song stimuli containing vocals to children with ASD (including low functioning ones) and aged matched controls. While brain activations were found to be different between the children with ASD and TD during the spoken condition, they turned out to be comparable when speech was delivered in a sung format (Lai et al., 2012). Another study found comparable brain activation patterns and preserved fronto-temporal connections between children with ASD and aged matched controls during perception of sung but not spoken words (Sharda et al., 2015). Most of the experiments reviewed here have shown abnormalities in lateralization and aberrant functional activation during speech processing during infancy and toddlerhood similarly to what has been reported in older children and adults. However, none of them varied the mode of presentation of the speech sounds that were always presented in a spoken format. Interestingly, one experiment found that toddlers with ASD (aged 2 years) who preferred motherese speech signals (that is, a pattern of speech characterized by high-pitch intonations) exhibit similar ERP responses compared to aged match TD controls in a passive syllable discrimination task (Kuhl et al., 2005). In this later, toddlers with ASD who didn't preferred an analog speech signal had different ERP responses compared to TD toddlers. It will be instrumental to detect how early the mode of communication impacts human speech processing in infants and toddlers with ASD and in at risk population as preliminary results now suggest that sung over spoken speech might effectively improve socio-communicative behaviors in toddlers with ASD at least (Paul et al., 2015).

Another final important aspect that has to be considered besides the various impairments in the development of speech processing highlighted by the relevant neuroimaging literature we reviewed here, is that functional and structural anomalies have also been reported at earlier stages of the auditory processing system in autism. Some studies highlight functional abnormalities within low level primary sensory auditory pathways in adults with ASD (Dinstein et al., 2012; Haigh et al., 2015). A recent experiment points toward the presence of maturational differences in the development of primary/secondary auditory areas in children with ASD aged between 6 and 14 years (Edgar et al., 2015). The presence of abnormal auditory brainstem response in newborns/infants (tested between 0 and 3 months) and toddlers (tested between 1.5 and 3.5 years) later diagnosed with ASD has also been demonstrated (Miron et al., 2015). Further work is thus required to understand how these impairments in the early stages of the auditory processing system might impinge on the development of speech and language processing in autism.

## Author contributions

HS and MS wrote the paper, read, and approved the final manuscript.

### Conflict of interest statement

The authors declare that the research was conducted in the absence of any commercial or financial relationships that could be construed as a potential conflict of interest.
